# A Binocular Information Source for Size Perception

**DOI:** 10.3389/fpsyg.2017.02078

**Published:** 2017-12-04

**Authors:** Nam-Gyoon Kim

**Affiliations:** Department of Psychology, Keimyung University, Daegu, South Korea

**Keywords:** size distance invariance hypothesis, size distance paradox, metric information, size perception, binocular information source

## Abstract

For too long, size perception research has been guided by the size distance invariance hypothesis. Although research to validate this hypothesis has been largely inconclusive, the hypothesis has endured, perhaps in part because alternative information sources for size perception were lacking. Here, I propose a binocular information source for size perception. The model, drawing on the binocular geometry of viewing a physical extent, is expressed solely in terms of four angular measures and interocular distance, with the explicit exclusion of egocentric distance information. Thus, the proposed information source, if utilized by the binocular system, should be able to augment the few existing sources of information for size perception (e.g., familiar size, texture gradient, and horizon ratio).

## Introduction

For successful encounters with our surrounding surfaces it is essential that we perceive the sizes of various objects accurately. Consider, for example, picking up an object. Two fingers suffice to pick up a cherry but not an apple. However, the grip needed to handle an apple must be readjusted to handle a melon. Consider an even simpler case, shaking hands, a universally accepted ritual of greetings. Even in this case, the opening of the hand must be adjusted to conform to the counterpart’s hand size. Clearly, shaking hands with a 5-year-old child differs from shaking hands with a female adult; and the latter differs from shaking hands with a 250-pound male adult.

Another, but equally important, aspect of size perception involves being able to perceive various sizes of gaps in the environment. To weave through a crowded mall, gaps that can be negotiated must be differentiated reliably from those that should be avoided. Consider an even more challenging case of driving in the narrow streets of any European city. Oftentimes, the cars are parked along the sides of an already tight street. The opposing drivers, however, somehow manage the situation, sometimes barely squeezing by each other while at other times letting the oncoming car go first. Actually, size perception is engrained in virtually every aspect of our daily activities in one way or another. Yet, we cope with these challenges with little effort and with precision. How do we accomplish these tasks so easily?

**FIGURE 1:**
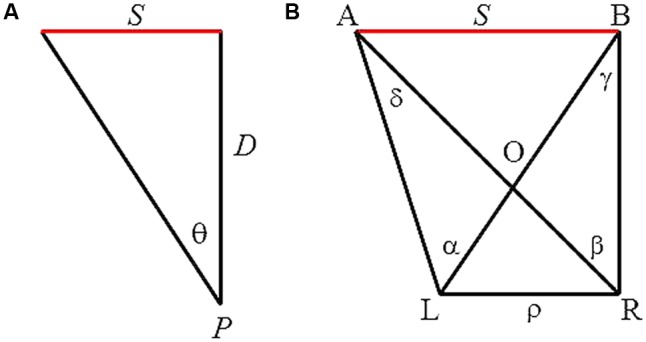
**(A)** Monocular geometry depicting the SDIH. An object of size *S* is at a distance *D* from an observation point, *P*, thus subtends a visual angle 𝜃. **(B)** Binocular geometry of viewing a line segment *AB*. L and R refer to the left and right eye, respectively, and hence, ρ the interocular distance. A and B are the two end points of the line segment. α and β are visual angles subtended by *AB* with respect to each eye, whereas γ and δ are binocular parallaxes of each end-point of the segment with respect to the two eyes.

Attempts to account for this perceptual capacity have a long history that includes some of the best minds in science: Euclid, Ptolemy, Alhazen, Descartes, Berkeley, Helmholtz, and many more (Hatfield, 2002). With the exception of a few authors (e.g., Berkeley), however, most assumed that size perception was based on the simple geometry of a right triangle (**Figure 1A**). Note that the two sides of the triangle, *S* and *D*, are inversely related to the angle, 𝜃, through a trigonometric relation, tan 𝜃 = *S*/*D*. When extended to perception, this geometric relation between *S* and *D* for a given angle 𝜃 forms a universally accepted law of visual perception, the size-distance invariance hypothesis (SDIH). The hypothesis states that the visual angle (𝜃) subtended by an object determines a unique ratio of the perceived size of the object (*S*′) to its perceived distance (*D*’), that is, tan 𝜃 = *S*’/*D*’ (Kilpatrick and Ittelson, 1953; Epstein et al., 1961).

The number of combinations between size and distance for any given angle is infinite. Yet, it is primarily perceived size, not perceived distance, to which the SDIH hypothesis is applied. This arises from the fact that so few sources of information have been identified to account for the perception of size (e.g., familiar size and relative size), compared to distance (Haber and Levin, 2001). Indeed, this disparity between candidate information sources for size and distance is rather puzzling given the more than 2500 years of probing into this issue. Moreover, the efficacy of the few information sources that have been identified for size perception, is questionable. For example, apart from its limited applicability to familiar objects only, familiar size has been shown to be more effective as an information source for distance than for perceived size (Gogel, 1977).

Hence, the perceived size of an object is thought to be determined by both the visual angle the object subtends and its perceived distance, that is, *S*’ = *D*’ tan 𝜃. Conceived this way, objective size becomes a property that is not perceived directly, but is derived indirectly via visual angle augmented by perception or knowledge of the object’s distance. For Helmholtz, this derivation, namely, calibrating visual angle by taking perceived distance into consideration, was an inferential or judgmental process of which the perceiver was unconscious of and thus was referred to as *unconscious inference*. The view that visual perception is mediated by unconscious inferences, eventually became the overriding theme in perception (see, for example, Rock, 1983; Epstein, 1995).

Numerous attempts have been made to validate the SDIH. The results, however, have been inconclusive at best and contradictory at worst (Heinemann et al., 1959; Foley, 1980; Sedgwick, 1986; Collewijn and Erkelens, 1990; Brenner and van Damme, 1998; Haber and Levin, 2001; Kim et al., 2016; but see Kaufman et al., 2006, for evidence in support of the SDIH). A classic demonstration of the contradictory results is the study reported by Heinemann et al. (1959). These authors reported decrease in apparent size with increase in the angle of convergence, consistent with the SDIH. However, reported distance of a target increased with increased convergence, contradicting the SDIH. Specifically, the target that appeared smaller was judged as farther away, and the target that appeared larger was judged as closer. These anomalies are collectively known as the *size-distance paradox* (Gruber, 1954; Ono et al., 1974; Kim, 2012; see Ross, 2003, for a review).

Despite contrary evidence, the SDIH has endured. Gillam (1998) speculates that the simplicity with which the hypothesis is portrayed, that is, as a geometric relationship between size and distance given a visual angle, may have contributed to its survival. Seeing the conflicting evidence arising from research directed at the SDIH, Gillam (1995) questioned whether size is a quantity derived from distance information, as contended by the SDIH, or a primary perceptual quality like motion. The latter has remained a conjecture, primarily due to the lack of suitable information sources for size perception.

Here I present a binocular source of information for the perception of size. This will add to the number of potential information sources for size perception.

## A Binocular Source of Information for Size Perception

**Figure 1B** depicts the geometry involved in binocular viewing of a line segment (this is the same geometry depicted in **Figure 1A** but viewed with two eyes). L and R refer to the left and right eye, respectively, and hence, ρ, the interocular distance; whereas A and B are the two end points of the line segment. α and β are visual angles subtended by *AB* with respect to each eye, whereas γ and δ are binocular parallaxes of each end point of the segment with respect to the two eyes. From this geometry, the frontal size *S* (i.e., the linear extent *AB*) can be expressed as follows:

Let *O* be the point of intersection of lines *AR* and *BL*, 𝜀 and η angles ∠OBA and ∠OAB, respectively. By applying the Sine Formula to the triangles ΔAOB and ΔLOR, respectively, we obtain

(1) $$\begin{array}{c} \frac{\mathrm{AO}}{\sin\;\varepsilon }=\frac{\mathrm{BO}}{\sin\;\eta } \end{array}$$

(2) $$\begin{array}{c} \frac{\mathrm{RO}}{\sin\;\varepsilon }=\frac{\mathrm{LO}}{\sin\;\eta }\;\left(2\right) \end{array}$$

By rearranging (1) and (2), we obtain

(3) $$\begin{array}{c} \frac{\mathrm{BO}}{\mathrm{AO}}=\frac{\mathrm{LO}}{\mathrm{RO}}\;\left(3\right) \end{array}$$

By applying the Sine Formula to the triangles 1AOL and 1BOR, respectively, we obtain

(4) $$\begin{array}{c} \frac{\mathrm{AO}}{\sin\;\alpha }=\frac{\mathrm{LO}}{\sin\;\delta } \end{array}$$

(5) $$\begin{array}{c} \frac{\mathrm{BO}}{\sin\;\beta }=\frac{\mathrm{RO}}{\sin\gamma } \end{array}$$

Dividing (5) by (4) gives the equation

(6) $$\begin{array}{c} \frac{\mathrm{BO}}{\mathrm{AO}}=\left(\frac{\sin\;\beta }{\sin\;\alpha }\frac{\sin\;\delta }{\sin\;\gamma }\right)\frac{\mathrm{RO}}{\mathrm{LO}} \end{array}$$

From (3) and (6), we obtain

LO=1sin αsin βsin γsin δRO

We also rearrange (5) in terms of *BO* to obtain

BO=sin βsin γRO

From two similar triangles ΔAOB and ΔLOR, *AB* can be obtained from

(7) $$\begin{array}{c} \mathrm{AB}=\rho \frac{\mathrm{BO}}{\mathrm{LO}} \end{array}$$

By substituting *BO* and *LO* into (7) and with further simplification, we obtain

(8) $$\begin{array}{c} \mathrm{AB}=\rho \sqrt{\frac{\sin\;\alpha }{\sin\;\delta }\frac{\sin\;\beta }{\sin\;\gamma }} \end{array}$$

The model is expressed solely in terms of four angular measures and interocular distance, with the explicit exclusion of egocentric distance information. Nevertheless, any frontal size can, in principle, be perceived binocularly based on the model provided that the visual system can access its interocular distance, which strong evidence suggests it can (e.g., Cutting and Vishton, 1995). In fact, for convergence angle to be utilized as a distance cue, it must be scaled by an observer’s interocular distance. To the extent that convergence incorporates the interocular distance, so does the proposed binocular information source. Thus, the proposed model, if utilized by the binocular system, should be able to augment the few existing sources of information for size perception (e.g., familiar size, texture gradient, and horizon ratio).^1^

Some additional features also stand out. Note that the model can provide a metric basis for an object’s size for the binocular system. Most sources of spatial information identified to date are relative, that is, they provide ordinal but not absolute metric information (see Kaufman, 1974, for further details).^2^ A metric source of information makes the proposed model even more unique. ^3^

The fact that the model is based on binocular information further enhances its utility. Of the many sources of spatial information identified to date, all are monocular except for convergence and binocular disparity. This is appalling considering that the two modes of visual perception may be fundamentally different-especially at short distances. Indeed, this was the conclusion da Vinci reached when he realized that even the best painting, that is, one in which light, contours, color and shadows of the object are perfectly represented, can never depict accurately the relief that occurs in natural objects (see Wade et al., 2001, for further details). Yet, the research on binocular vision has been largely dependent upon a set of discoveries and premises made to describe monocular phenomena, in particular, the SDIH.

Nevertheless, certain qualifications should also be recognized that limit the proposed variable as the source of information for binocular size perception. First, binocular depth perception has been shown to be effective only at short distances, limited probably to 2 m or less (see Ono and Comerford, 1977, for review). The proposed variable may be subject to a similar stereoscopic limit.

Second, the proposed information source is applicable only to the horizontal extent of a line segment. However, this limitation may actually strengthen its validity. Wraga (1999; see also Dixon et al., 2000) demonstrated that human observers utilize the horizon ratio to scale height judgments, but its efficacy with respect to width judgments is minimal. Based on this finding, Wraga (1999) concluded that perceptual capacity to estimate the vertical extents of objects appears to be largely dissociated from that for horizontal extents, a possibility anticipated by Gillam (1995; see Figure 13). Thus, an information source such as that proposed here is needed to provide a perceptual basis for horizontal size judgments.

Another limitation comes from the fact that the proposed model was derived assuming that the line segment lies in the frontal–parallel plane. Hence, the model can only provide an accurate description of frontal size. In size perception literature, size typically is defined as a linear extent in a frontal-parallel plane (Ono and Comerford, 1977). Thus, for the SDIH to apply, the linear extent *S* depicted in **Figure 1A** must lie perpendicular with respect to the line of regard. Only under this simplified situation can the trigonometric relation *S* = *D* tan 𝜃 be utilized to describe perceived size (see Sedgwick, 1986, for details).

The issue remains as to how the visual system perceives the lengths of slanted objects. Patently, objects can be slanted and/or tilted away from the frontal-parallel plane. These changes, however, alter the appearance of the objects; and additional information, such as slant and/or tilt angle, is assumed to be necessary to recover shape and size (i.e., shape constancy). The question can be raised as to whether the perceived size of a slanted object is veridical, perhaps with some type of compensation for its slanted angle, or is distorted in conformity with its projected view. Evidence suggests that human observers are poor at judging the lengths of slanted line segments and, by extension, the shapes of objects (Todd et al., 1995; Norman et al., 1996; Bingham et al., 2000; Bingham, 2005).

Norman et al. (1996) examined the perception of linear extents while varying their orientations from the frontal-parallel plane to the sagittal plane, concluding that frontal–parallel lengths (i.e., frontal size) were perceived differently than slanted lengths, including the special case of the lengths aligned along the sagittal plane or in-depth lengths (a linear extent lying along a sagittal plane is referred to as depth). Significantly, perception of frontal size was accurate, and remained so, across all viewing distances employed; whereas the perception of slanted lengths was distorted (or compressed), with the degree of distortion deteriorating with increasing viewing distance. Taken together, Norman et al.’s (1996) reports of accurate perception of frontal size and possible differences between the perception of frontal size and of slanted lengths are consistent with what the proposed information source entails, and thus provide further support for its validity.

In summary, mounting evidence suggests that the SDIH may not be a suitable scheme to describe the perception of size, let alone as a model for binocular size perception. The alternative model proposed here, by contrast, is a binocular source conveying absolute metric information. More importantly, the model assumes that the information for an object’s size is directly available in optical stimulation.

## General Discussion

For too long, size perception research has been dictated by the doctrine of size-distance invariance. Despite mounting evidence to the contrary, the doctrine has endured. Perhaps, as Gillam (1995) suspects, it is the simplicity of the geometry with which the hypothesis is portrayed (**Figure 1A**). Surprisingly, however, almost 25 centuries of research into size perception have resulted in virtually no candidate information source for size perception for the binocular visual system. A similar sentiment was expressed by Haber and Levin (2001) who, after failing to explain how their subjects arrived at accurate size estimations for unfamiliar objects, remarked:

All we can say is that they did not do it in the same way as they did for the distance estimations. This ignorance reflects a general ignorance about the perceptual variables underlying size perception. Most of the theoretical discussions about size perception appeal to familiarity (as do we) and ignore any other variables. But *there must be some others*, and *size perception theorists have to identify and demonstrate them*. (p. 1150; emphasis added)

I have proposed a potential source of information for size perception that can fill this void. The model draws on the binocular geometry of viewing a physical extent. Hence, this model qualifies as one of a few binocular sources of information that have been identified to date. Added significance is the fact that the model, unlike other sources of information for spatial vision that provide only relative information, can provide an absolute metric for size perception. More importantly, the information for an object’s size is directly available in optical stimulation, even in the absence of egocentric distance information. With perceptions of size and distance no longer tied together as in the SDIH, changes in one percept (i.e., perceived size) bear little influence on its counterpart (i.e., perceived distance).

As Haber and Levin (2001) note, research on space perception over the last 200 years has placed much less emphasis on size perception than on distance perception. It is hoped that the present study will alter that balance. Clearly, further research is needed to validate the utility of this variable for the binocular visual system. Nonetheless, the model is promising, considering the fact that so few sources of information have been identified to date that can provide metric bases for binocular space perception.

## Author Contributions

The author confirms being the sole contributor of this work and approved it for publication.

## Conflict of Interest Statement

The author declares that the research was conducted in the absence of any commercial or financial relationships that could be construed as a potential conflict of interest. The reviewer JG and handling Editor declared their shared affiliation.

## Footnotes

1 To exploit binocular convergence to determine the distance between the observer and the fixation point, the visual system must perform a trigonometric calculation involving interocular distance and the angle of convergence. Levine (2004, p. 236) notes that “of course, our visual systems do not perform a trigonometric calculation, but it is reasonable to expect to learn the distance significance of the convergence signal”. Admittedly, the proposed model is even more complex as it is composed of a combination of several trigonometric terms. Nevertheless, it is conceivable that this signal can be picked up by the visual system. Indeed, perceptual systems are thought to be extremely “smart” such that they are capable of detecting higher order invariants specifying salient dimensions of the environment (Runeson, 1977). A case in point is τ ^⋅^ (or “tau-dot”) corresponding to the time derivative of the inverse of the relative rate of optical angle subtended by an approaching object specifying the impact of collision with the object (Lee, 1976). Research further confirmed that human observers are, not only sensitive to this optical variable (Kim et al., 1993; Andersen et al., 1999; Kim, 2015), but also use it in the visual control of braking (Yilmaz and Warren, 1995; Rock and Harris, 2006).

2 With respect to the distinction between spatial information being absolute and relative, according to Kaufman (1974, p. 222), “Absolute distance refers to the distance between the observer and an object—i.e., the observer is at point zero, and objects are particular linear distances from point zero. Relative distance refers to the distance between objects independent of the distance from the observer to one of the objects”. Consequently, Kaufman notes that, by virtue of all cues to depth being relative, that is, “indicating that one object is more or less distant from an observer than is another object, … the distance cues cannot be used in a simple computation of size (as implied by Helmholtz), since they do not indicate absolute distance. It would first be necessary for the relative distance cues to be used in a computation of absolute distance, and we do not know how this might be done” (p. 349). Then, the question can be raised as by Cutting and Vishton (1995): “How is it that we perceive layout with near-metric accuracy when none of these sources yield metric information about it?” (p. 109). Whereas, following Gibson (1950, 1979/1986; see also Michaels and Carello, 1981) I search for higher order invariants specifying surrounding layout, e.g., the horizon ratio, texture gradients, optical flow, etc., to account for this capacity, other researchers seek for solutions by way of combining multiple information sources (Cutting and Vishton, 1995; Landy et al., 1995, to name a few). Further discussion of this issue, however, goes beyond the scope of this manuscript and readers are referred to the vast literature on cue integration.

3 Kaufman (1974, p. 257) points out that “of all the cues we have considered so far, convergence may well be the only one to give absolute distance information. … the Euclidean cues, relative brightness, interposition, the kinetic cues, and even accommodation can inform only about relative distance”. However, the horizon ratio should be considered another source of metric information with eye height as a scale factor (see Sedgwick, 1986; Wraga, 1999).
